# A comparison of skeletal, dentoalveolar and soft tissue characteristics in white and black Brazilian subjects

**DOI:** 10.1590/S1678-77572010000200007

**Published:** 2010

**Authors:** Lívia Maria Andrade de FREITAS, Karina Maria Salvatore de FREITAS, Arnaldo PINZAN, Guilherme JANSON, Marcos Roberto de FREITAS

**Affiliations:** 1 DDS, MSc, PhD, Graduate student, Department of Pediatric Dentistry, Orthodontics and Community Health, Bauru School of Dentistry, University of São Paulo, Bauru, SP, Brazil.; 2 DDS, MSc, PhD, Professor at Ingá Faculty of Dentistry (UNINGÁ), Maringá, PR, Brazil.; 3 DDS, MSc, PhD, Associate Professor, Department of Pediatric Dentistry, Orthodontics and Community Health, Bauru School of Dentistry, University of São Paulo, Bauru, SP, Brazil.; 4 DDS, MSc, PhD, Full Professor, Department of Pediatric Dentistry, Orthodontics and Community Health, Bauru School of Dentistry, University of São Paulo, Bauru, SP, Brazil.

**Keywords:** Ethnic groups, Cephalometry, Normal values

## Abstract

**Objective:**

This study aimed to compare skeletal, dentoalveolar and soft tissue characteristics in white and black Brazilian subjects presenting normal occlusions.

**Material and Methods:**

The sample comprised the lateral cephalograms of 106 untreated Brazilian subjects with normal occlusion, divided into two groups: Group 1- 50 white subjects (25 of each gender), at a mean age of 13.17 years (standard deviation 1.07); and Group 2- 56 black subjects (28 of each gender), at a mean age of 13.24 years (standard deviation 0.56). Variables studied were obtained from several cephalometric analyses. Independent t tests were used for intergroup comparison and to determine sexual dimorphism.

**Results:**

black subjects presented a more protruded maxilla and mandible, a smaller chin prominence and a greater maxillomandibular discrepancy than white subjects. Blacks presented a more horizontal craniofacial growth pattern than whites. Maxillary and mandibular incisors presented more protruded and proclined in black subjects. The nasolabial angle was larger in whites. Upper and lower lips were more protruded in blacks than in whites.

**Conclusions:**

The present study found a bimaxillary skeletal, dentoalveolar and soft tissue protrusion in black Brazilian subjects compared to white Brazilian subjects, both groups with normal occlusion. Upper and lower lips showed to be more protruded in blacks, but lip thickness was similar in both groups.

## INTRODUCTION

It is known that a single standard of cephalometric variables is not appropriate for application to diverse racial and ethnic groups, and that normative data of cephalometric measurements are essential to precisely determine the degree of variation from normal^14,21^.

Orthodontic treatment must be in equilibrium with the normal growth process to be effective and stable and to compensate for unpleasant facial patterns. The impact of treatment on the face has been constantly questioned. The age and the race became indispensable features^18^. The cephalometric norms are not applicable to all patients because of the racial characteristics and the miscegenation, bringing the need for specific cephalometric standards to different ethnic groups^1^.

Cephalometric norms of different ethnic groups must be interpreted with caution. American blacks are an admixture not only of the different races in the United States, but also come from different parts of Africa^7^. In the same way, Brazilian blacks had their origin mainly from the African coast, where Bantu population is prevalent. Some studies demonstrated significant cephalometric differences between South African, American blacks and whites, due to interracial and intraracial variations in morphological characteristics^2,5,11,13^. The black subjects generally present a dental camouflage to compensate an anteroposterior discrepancy of skeletal bases, providing a good facial balance^5^. Enlow, et al.^13^ (1982) affirmed that, in Class I cases, craniofacial patterns are differentiated among blacks and whites. In blacks, the mandible develops downwards in a greater proportion than in whites. However, other studies found a bimaxillary protrusion characterized by dentoalveolar flaring of both maxillary and mandibular teeth with resultant protrusion of the lips and convexity of the face in black subjects^4,11,15,16^.

Considering the factors involved in ethnic facial features, it becomes important to study the Brazilian population considering the respective somatic traits. The present study aimed to cephalometrically compare skeletal, dentoalveolar and soft tissue characteristics in two distinct ethnic groups: black and white young Brazilian subjects with normal occlusion. The tested null hypothesis was that the cephalometric characteristics of black and white young Brazilian subjects with normal occlusion are similar.

## MATERIAL AND METHODS

The sample comprised the lateral cephalograms of 106 white and black untreated young Brazilian subjects presenting normal occlusion and well-balanced faces. The whole sample was obtained from the Growth Center at Bauru Dental School, University of São Paulo, and divided into two groups: Group 1 included 50 white subjects (25 of each gender) at a mean age of 13.17 years (standard deviation 1.07, range from 11.40 to 14.90), and group 2 included 56 black subjects (28 of each gender) at a mean age of 13.24 years (standard deviation 0.56, range from 12.08 to 14.33). All subjects presented all permanent teeth up to the second molars and normal occlusion, i.e., normal molar and canine relationship, absence of crowding and crossbites, normal overjet and overbite, wellbalanced face and without previous history of orthodontic treatment. Their data were collected some years ago when there were lighter restrictions on human studies.

The subjects of both ethnic groups were selected as pure as possible from the same geographic boundary, and the parents of each correspondent subject were from the same ethnic group. The Brazilian black subjects had their origin mainly from the African coast, where Bantu population is prevalent. Brazilian whites were Mediterranean descents.

It is important to study the population characteristics and the origin of the Brazilian ethnic groups, analyzing the respective somatic traits. Other relevant factor is the historic mixture of innumerous populations and races in America, which hinders the biological definition of each group^16^. The miscegenation in Brazil among the Portuguese, the indigenes and black individuals resulted in the formation, since the early times of History, of a diversified population. Each one of the three basic groups is far from representing a pure ethnic group. By the geographic origin, one can have an idea of the racial affiliation of the imported individuals of the black group. In the African coast the Bantus are predominant, who were selected by the present sample, formed by the mixture of nigricians and paleonegroids, divided in occidental, oriental and meridional, with great or less influence. The Brazil stands as one of the few American countries that received African people of all origins. Three regions of Africa, the west, center-west and southeast coasts contributed with slave workers to Brazil until 1850^29^.

Regarding the cephalic index and stature, the following ethnic groups were distinguished in the Negroid group:

1. The Nigrician, with high percentage of tall and dolichocephalic individuals; concentrated in Sudan and Guinea;

2. The Paleonegroid, with high percentage of short and mesocephalic individuals; concentrated in the forest regions of Congo, Senegal and Angola;

3. The Nilotic, with really tall and dolichocephalic individuals; dispersed in regions of High Nilo and great lakes;

4. The Khoisan, with high percentage of short and mesocephalic individuals; dispersed in South Africa, as well as the forest and desert regions.

**Figure 2:**
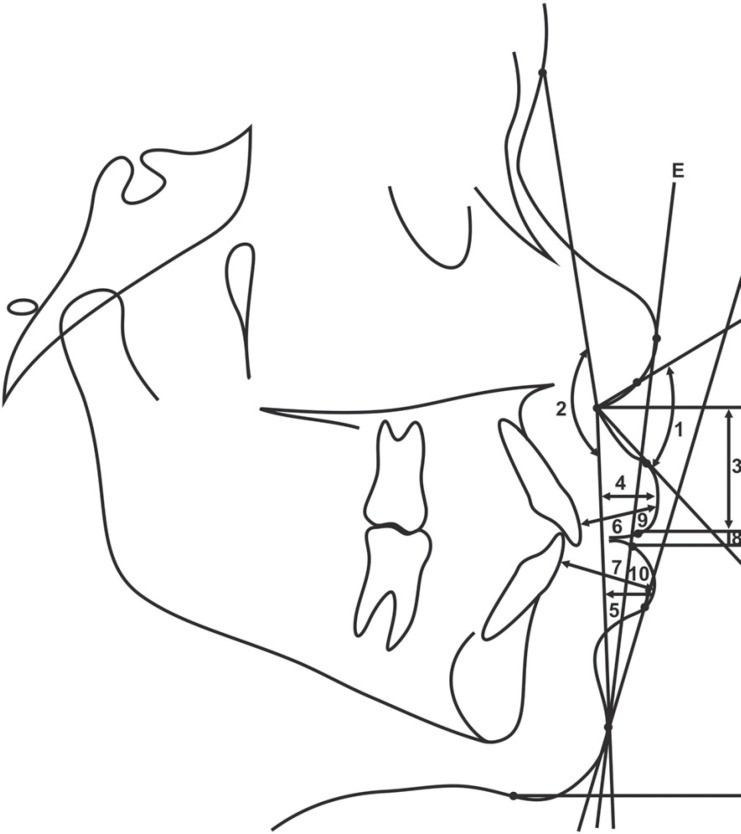
Less usual cephalometric variables: 1. Nasolabial angle (^0^); 2. Soft tissue convexity (^0^); 3. Upper lip length (mm); 4. Upper lip protrusion (mm); 5. Lower lip protrusion (mm); 6. Upper lip thickness (mm); 7. Lower lip thickness (mm); 8. Interlabial gap (mm); 9. Upper lip-E (mm); 10. Lower lip-E (mm); Line E (Ricketts esthetic plane)

The cephalometric tracings and landmark identifications were performed on acetate paper by a single investigator (LMAF) and digitized (Numonics AccuGrid XNT, model A30TL.F- Numonics Corporation, Montgomeryville, Pa). These data were then stored on a computer and analyzed with Dentofacial Planner 7.02 (Dentofacial Planner Software Inc., Toronto, Ontario, Canada), which corrected the magnification factor of the radiographic images (6% for both groups). Skeletal, dentoalveolar and soft tissue cephalometric measurements are shown in Figure 1 and less usual variables are shown in Figure 2.

**Figure 1:** Definitions of abbreviations of the cephalometric variables evaluated in this study

**SNA (º)-** Angle formed by line S-N and line N-A.
**A-Nperp (mm)-** Linear distance from point A to the line perpendicular to Frankfort plane passing through point N.
**SNB (º)-** Angle formed by line S-N and line N-B.
**Co-Gn (mm)-** Linear distance between the points condylion and gonion.
**P-Nperp (mm)-** Linear distance from point P to the line perpendicular to Frankfort plane passing through point N.
**P-NB (mm)-** Linear distance from point P to the line N-B.
**ANB (º)-** Angle formed by line N-A and line N-B.
**Convexity (NAP) (°)-** Angle formed by line N-A and line A-P.
**Wits (mm)-** Linear distance between the projections of points A and B on occlusal plane.
**FMA (º)-** Angle formed by Frankfort plane and mandibular plane (GoMe).
**SN.GoGn (º)-** Angle formed by line S-N and line Go-Gn.
**SN.Ocl (º)-** Angle formed by line S-N and occlusal plane.
**1.NA (º)-** Angle formed by maxillary incisors long axis and line N-A.
**1-NA-** Linear distance from the most anterior point of the crown of maxillary incisor to line N-A.
**1.NB (º)-** Angle formed by mandibular incisors long axis and line N-B.
**1-NB-** Linear distance from the most anterior point of the crown of mandibular incisor to line N-B.
**Mentolabial sulcus-** Longer distance from the mentolabial sulcus to line formed by the most anterior point of lower lip and the soft tissue pogonion.
**Nasolabial angle (º)-** Angle formed by a line from the lower border of the nose to one representing the inclination of the upper lip.
**Soft tissue convexity (º)-** Angle formed between the lines from soft tissue glabella to subnasale and pogonion.
**Upper lip length (mm)-** Linear distance between the subnasale point and the most inferior point on the vermilion of the upper lip.
**Upper lip protrusion (mm)-** Linear distance between upper lip anterior point and subnasale-pogonion line.
**Upper lip thickness (mm)-** Linear distance between upper lip anterior point and the most anterior point of the buccal surface of maxillary incisor.
**Lower lip protrusion (mm)-** Linear distance between lower lip anterior point and subnasale-pogonion line.
**Lower lip thickness (mm)-** Linear distance between lower lip anterior point and the most anterior point of the buccal surface of mandibular incisor.
**Interlabial gap (mm)-** Linear distance between the most inferior point on the vermilion of the upper lip to the most superior point on the vermilion of the lower lip.
**Lower lip-E (mm)-** Linear distance between the lower lip anterior point and line E
**Upper lip-E (mm)-** Linear distance between the upper lip anterior point and line E (esthetic plane by Ricketts).

The mean and standard deviation (SD) for the ages and for each variable were calculated for both groups. Normal distribution was verified by the Kolmogorov-Smirnov test. The results of the tests were non-significant for all variables. Therefore, intergroup comparisons were performed by independent t tests. All statistical analyses were performed on Statistica software (Statistica for Windows 6.0; Statsoft, Tulsa, Okla), with a level of significance of 5%.

After 1-month interval from the first measurement, thirty randomly selected cephalograms were retraced and re-measured by the same examiner (LMAF). Random errors were calculated according to Dahlberg’s formula^10^ (Se^2^= Σd^2^/2n) where Se^2 ^ is the error variance and d is the difference between the two determinations of the same variable, and the systematic errors were evaluated with dependent t tests^19^, for p<0.05.

## RESULTS

The random errors varied from 0.40 mm (Wits) to 0.92 mm (LL-E) and from 0.50º (SN.GoGn) to 0.97º (ST convexity). Only one angular variable (SN.Ocl) and two linear variables (1-NB and UL thickness) presented statistically significant systematic errors. From the 28 measured evaluated, only two presented systematic errors: SN.Ocl and 1-NB (Table 1). These results demonstrated that 92.54% of the studied variables presented precision and coherence. These errors were comprehensible, because it is known that there is great variation in the determination of the mandibular incisor root apex.

**Table 1:** Casual and systematic errors between the 1^st^ and 2^nd^ measurements

	**1^st^ measurement**		**2^nd^ measurement**				
**Variables**	**Mean**	**SD**	**Mean**	**SD**	**N**	**Dahlberg**	**P**
				**Maxillary component**			
SNA (º)	84.91	4.29	84.80	4.43	30	0.83	0.218
A-Nperp (mm)	1.75	3.88	1.84	3.82	30	0.42	0.308
				**Mandibular component**			
SNB (º)	81.39	3.62	81.37	3.68	30	0.71	0.883
Co-Gn (mm)	110.02	6.28	109.97	6.11	30	0.46	0.614
P-Nperp (mm)	-2.33	6.60	-2.16	6.73	30	0.53	0.085
P-NB (mm)	0.49	1.61	0.54	1.65	30	0.41	0.580
				**Maxillomandibular relationship**			
ANB (º)	3.51	2.38	3.39	2.46	30	0.60	0.345
Convexity (NAP) (º)	6.87	5.63	6.99	5.61	30	0.89	0.257
Wits (mm)	-0.67	2.91	-0.56	3.02	30	0.40	0.231
				**Vertical component**			
FMA (º)	24.58	4.32	24.60	4.32	30	0.75	0.837
SN.GoGn (º)	31.23	3.99	31.25	3.92	30	0.50	0.820
SN.Ocl (º)	14.03	3.55	14.24	3.50	30	0.93	0.020 *
				**Dentoalveolar component**			
1.NA (º)	23.54	6.72	23.50	6.84	30	0.83	0.667
1-NA (mm)	5.11	3.28	5.24	3.79	30	0.74	0.475
1.NB (º)	31.07	7.55	31.08	7.72	30	0.61	0.928
1-NB (mm)	6.66	2.52	6.87	2.64	30	0.46	0.032 *
				**Soft tissue component**			
Mentolabial sulcus (mm)	3.85	0.97	3.76	1.07	30	0.44	0.362
Nasolabial angle (º)	96.89	14.39	96.74	14.28	30	0.86	0.123
ST convexity (º)	14.12	6.40	14.11	6.44	30	**0.97**	0.922
UL length (mm)	24.95	3.15	24.85	3.36	30	0.42	0.261
UL protrusion (mm)	4.91	2.64	5.10	2.65	30	0.50	0.060
UL thickness (mm)	12.56	1.69	12.13	1.90	30	0.79	0.006 *
LL protrusion (mm)	4.64	3.37	4.65	3.49	30	0.43	0.888
LL thickness (mm)	14.46	1.27	14.36	1.24	30	0.59	0.292
Interlabial gap (mm)	1.30	1.51	1.47	1.65	30	0.49	0.063
LL-E (mm)	1.45	3.76	1.52	3.80	30	0.57	0.344
UL-E (mm)	-1.97	3.23	-1.56	3.45	30	**0.92**	0.083

* Statistically significant for *P* <.05. SD = standard deviation

Black subjects presented a significantly more protruded maxilla and mandible and a greater maxillomandibular anteroposterior discrepancy than white subjects which had a more vertical growth pattern. Chin prominence was larger in whites. Facial convexity was greater in blacks than in whites. The maxillary and mandibular incisors were more protruded and proclined in black subjects. The nasolabial angle was greater in whites than in blacks. The upper lip was longer and both upper and lower lips were significantly more protruded in blacks in relation to white subjects. And all of these differences were statistically significant (Table 2).

**Table 2:** Means and standard deviations for all variables in the two groups and results of independent t test

	**Group 1 White subjects N=56**		**Group 2 Black subjects N=50**		
**Variables**	**Mean**	**SD**	**Mean**	**SD**	**P**
Age (years)	13.17	1.07	13.24	0.56	0.632
			**Maxillary component**		
SNA (º)	81.68	2.89	86.95	3.89	0.000 *
A-Nperp (mm)	-0.15	2.73	4.07	3.47	0.000 *
			**Mandibular component**		
SNB (º)	78.83	2.73	82.95	3.52	0.000 *
Co-Gn (mm)	110.97	5.41	108.61	5.97	0.036 *
P-Nperp (mm)	-4.22	5.44	0.80	6.06	0.000 *
P-NB (mm)	1.41	1.46	-0.22	0.96	0.000 *
			**Maxillomandibular relationship**		
ANB (º)	2.82	2.27	3.99	2.17	0.007 *
Convexity (NAP) (º)	4.60	4.89	8.47	4.88	0.000 *
Wits (mm)	-0.62	2.76	-1.02	2.23	0.418
			**Vertical component**		
FMA (º)	25.32	4.40	23.48	4.53	0.036 *
SN.GoGn (º)	33.01	3.98	30.54	4.42	0.003 *
SN.Ocl (º)	15.97	3.81	13.44	3.43	0.000 *
			**Dentoalveolar component**		
1.NA (º)	21.59	5.75	24.92	5.43	0.002 *
1-NA (mm)	3.62	2.37	6.06	2.76	0.000 *
1.NB (º)	24.64	4.78	35.99	5.92	0.000 *
1-NB (mm)	4.37	1.99	8.14	2.23	0.000 *
			**Soft tissue component**		
Mentolabial sulcus	3.65	0.99	4.02	0.96	0.056
Nasolabial angle (º)	104.68	10.20	89.31	12.44	0.000 *
ST convexity (º)	14.88	5.91	12.98	4.89	0.074
UL length (mm)	24.10	2.37	25.95	2.84	0.000 *
UL protrusion (mm)	3.06	1.53	6.59	2.06	0.000 *
UL thickness (mm)	12.76	1.62	12.87	1.76	0.729
LL protrusion (mm)	1.58	2.04	6.25	2.12	0.000 *
LL thickness (mm)	14.46	1.12	14.66	1.39	0.420
Interlabial gap (mm)	0.90	0.84	1.30	1.43	0.088
LL-E (mm)	-1.96	2.32	3.51	2.32	0.000 *
UL-E (mm)	-4.23	2.08	0.16	2.59	0.000 *

* Statistically significant for *P* <.05. SD = standard deviation

## DISCUSSION

### Sample Selection

There are many studies in both black and white ethnic groups, but no one compares the skeletal, dentoalveolar and soft tissue characteristics in white and black Brazilians with normal occlusion. Furthermore, problems that can be identified when comparing cephalometric studies of white or black subjects are the cephalometric measurements used, differences in sample size and age, selection criteria, statistical methods, definitions of clinical normality, definitions of the black racial designation and variation in geographic distribution and origin of these two ethnic groups^16,28^.

This way, subjects of the two ethnic groups evaluated in this study were selected from the same geographic boundary, and parents of each subject must be from the same ethnic group. All sample presented normal occlusion and wellbalanced faces. Additionally, the groups were compatible regarding gender and age distribution (Table 2).

### Intergroup Comparison

#### Maxillary and mandibular components

Black subjects with normal occlusion presented statistically significant more protruded maxilla and mandible than white subjects with normal occlusion (Table 2). Several previous studies also found maxillary and mandibular prognathism in black subjects^2-4,11,17,22^. Anterior cranial base length can influence the anteroposterior position of nasion and therefore can affect the values of angles SNA and SNB, and this should be considered when comparing two different ethnic groups.^2,4^ Since black individuals present a shorter cranial base, increased values for the angles SNA and SNB could be expected^2,4,24^. The present study also found significant results for the variables A-Nperp and P-Nperp, confirming the bimaxillary skeletal prognathism of the black sample.

However, other studies did not find a statistically significant mandibular prognathism in black individuals, but the maxillary prognathism was also observed^5,8,20^. These controversies may be due to differences in ethnical origins of the samples.

Despite the greater mandibular protrusion observed in blacks, they presented smaller chin prominence when compared to whites, as indicated by P-NB (Table 2).

### Maxillomandibular relationship

The maxillomandibular relationship presented larger values for blacks in relation to whites and it is in agreement with most of the previous studies^3,5,11,12,20^ (Table 2). This difference in ANB angle can be explained by the differences in SNA and SNB angles. Even though the SNB angle was larger in blacks than in whites, it was not large enough to compensate for the large SNA angle, resulting in the larger ANB difference found for black subjects^11^. Following the same tendency as ANB, skeletal convexity (NAP) was greater in blacks than in whites (Table 2).

The wits appraisal did not show significant difference between black and white subjects. Some studies had reported that blacks tend to present shorter anterior cranial base, when compared to whites^2,4,24^. This way, relative to nasion it was expected that the maxilla (point A) and mandible (point B) were more anteriorly positioned in blacks than in whites. But, when the maxillomandibular relationship was evaluated in relation to the occlusal plane, there was no difference between blacks and whites, corroborating some previous studies^5,11,24^.

### Vertical components

Blacks presented a more horizontal craniofacial growth pattern than whites for all vertical component measurements. This result is in agreement with the results reported by Dandajena and Nanda^11^ (2003), when evaluating a Zimbabwean sample.

Some previous studies found that black Americans^6,9,12,28^ and Africans^2,4,20^ had a high Frankfort-mandibular plane angle (FMA). Differences from these studies to the present results emphasize the importance of different cephalometric norms for each ethnic group from distinct geographic origins.

### Dentoalveolar components

Regarding the dentoalveolar characteristics, black subjects presented more protruded and proclined maxillary and mandibular incisors than white subjects in all angular and linear incisor variables corroborating previous studies that found a bimaxillary dentoalveolar protrusion^2,4,5,11,12^ (Table 2). Nevertheless, some studies demonstrated only a greater labial inclination of the mandibular incisors and not for the maxillary incisors in blacks, in relation to whites^16,20^.

The black Brazilian subjects present greater tendency to present dental protrusion, when compared to whites, probably due to the greater African miscegenation in Brazil, in these individuals of African descent. This way, the greater maxillary skeletal prognathism compared to mandibular, as excessive buccal inclination and protrusion of the mandibular incisors, associated to a retropositioning of the chin, are the compensatory effects in order to maintain the incisal contact, in the Black group^11^.

The protrusion of the maxillary and mandibular incisors found in black individuals appears to compensate for the maxillary and mandibular prognathism, and for the deficient maxillomandibular relationship in order to maintain incisal contact^11^. Furthermore, this dental protrusion is more pronounced in mandibular incisors, compensating the smaller mandibular protrusion and chin prominence in this ethnic group.

### Soft tissue component

White subjects with normal occlusion presented a greater nasolabial angle than black subjects with normal occlusion, which presented greater upper lip length and protrusion and lower lip protrusion (Table 2). This indicates a greater soft tissue projection in blacks, as already mentioned previously ^2,12,16,26^.

In the present study, thickness of upper and lower lips was not found to differ between black and white groups. Most significant soft tissue measurements were the protrusion of upper and lower lips found in blacks when compared to whites, which reflected the protrusive pattern of skeletal and dental structures. These increased values for upper and lower lips protrusion reflect the bimaxillary dentoalveolar protrusion found in black individuals^2,4,11,12,20^, which does not mean that there is also a greater soft tissue thickness^17^, as demonstrated in the present results.

### Final Considerations

The esthetic facial lines and respective parameters differ in different ethnic groups, establishing individualized soft tissue measurements^26^. The compensatory dentoalveolar mechanisms provide a balanced face in distinct groups, different by age, race or gender^27^. Potentially orthodontic patients have a variety of profile preferences, which indicates a distinction in several facial characteristics within each ethnic group, and the contemporary concept of pleasant esthetics of the facial profile is even more subjective^21,23,25^.

The present study confirmed the bimaxillary skeletal, dentoalveolar and soft tissue protrusion observed in black subjects, which have been described by several authors^2,4,8,11,15,16,20,22^. This dentoalveolar protrusion found in blacks is more evident in the mandibular incisors, compensating the slightly smaller protrusion of the mandible and the smaller chin prominence in this ethnic group.

As expected, blacks showed greater upper and lower lip protrusion^2,12,16^. However, thickness of upper and lower lips was unexpectedly similar in both groups. This reaffirms that the greater soft tissue projection in blacks is actually a consequence of protruded maxillary and mandibular incisors.

## CONCLUSIONS

The null hypothesis was rejected, because black and white young Brazilian subjects with normal occlusion showed different cephalometric characteristics.

Black Brazilian subjects with normal occlusion presented a more protruded maxilla and mandible, a smaller chin prominence, a greater maxillomandibular discrepancy, a more horizontal craniofacial growth pattern and more protruded and proclined maxillary and mandibular incisors than white Brazilian subjects with normal occlusion. The nasolabial angle was larger in whites. Upper and lower lips were more protruded in blacks, but lip thickness was similar in both groups.
